# Enhancement of Lymphangiogenesis *In Vitro* via the Regulations of HIF-1**α** Expression and Nuclear Translocation by Deoxyshikonin

**DOI:** 10.1155/2013/148297

**Published:** 2013-04-22

**Authors:** Orawin Prangsaengtong, Jun Yeon Park, Akiko Inujima, Yoshiko Igarashi, Naotoshi Shibahara, Keiichi Koizumi

**Affiliations:** ^1^Department of Kampo Diagnostics, Institute of Natural Medicine, University of Toyama, Toyama 930-0194, Japan; ^2^Department of Biopharmacy, Faculty of Pharmacy, Srinakharinwirot University, Nakhonnayok 26120, Thailand

## Abstract

The objectives of this study were to determine the effects of deoxyshikonin on lymphangiogenesis. Deoxyshikonin enhanced the ability of human dermal lymphatic microvascular endothelial cells (HMVEC-dLy) to undergo time-dependent *in vitro* cord formation. Interestingly, an opposite result was observed in cells treated with shikonin. The increased cord formation ability following deoxyshikonin treatment correlated with increased VEGF-C mRNA expression to higher levels than seen for VEGF-A and VEGF-D mRNA expression. We also found that deoxyshikonin regulated cord formation of HMVEC-dLy by increasing the HIF-1**α** mRNA level, HIF-1**α** protein level, and the accumulation of HIF-1**α** in the nucleus. Knockdown of the HIF-1**α** gene by transfection with siHIF-1**α** decreased VEGF-C mRNA expression and cord formation ability in HMVEC-dLy. Deoxyshikonin treatment could not recover VEGF-C mRNA expression and cord formation ability in HIF-1**α** knockdown cells. This indicated that deoxyshikonin induction of VEGF-C mRNA expression and cord formation in HMVEC-dLy on Matrigel occurred mainly via HIF-1**α** regulation. We also found that deoxyshikonin promoted wound healing *in vitro* by the induction of HMVEC-dLy migration into the wound gap. This study describes a new effect of deoxyshikonin, namely, the promotion of cord formation by human endothelial cells via the regulation of HIF-1**α**. The findings suggest that deoxyshikonin may be a new drug candidate for wound healing and treatment of lymphatic diseases.

## 1. Introduction

Lymphangiogenesis is similar to angiogenesis and refers to the formation of lymphatic vessels from preexisting lymphatic vessels, which play an important role in tissue-fluid homeostasis, as a tissue drainage system, immunosurveillance, and absorption of dietary fat [1]. Dysfunction of lymphatic vessels leads to chronic edema and impairment of immune responses. In adult tissue, the induction of new lymphatic vessel growth also promotes inflammation, wound healing, and tumor metastasis to the lymph node [2]. The molecular mechanisms of angiogenesis and its treatment are already well known, whereas understanding of the functions and regulatory pathways of lymphangiogenesis and its treatment has been far less explored [1].

Vascular endothelial growth factors (VEGFs) are interesting inducers of lymphangiogenesis, because they are a highly specific mitogen for endothelial cells[3] and transcriptional factors; hypoxia-inducible factor-1 (HIF-1), which is composed of two subunits, HIF-1alpha (HIF-1*α*) and HIF-1beta (HIF-1*β*) [4, 5], can modulate VEGF gene expression [6–8]; however, the role of these regulators in the lymphangiogenesis process is poorly understood. 

Shiunko is a typical Kampo drug ointment (a traditional botanic formula) used for the treatment of burns and wounds in Japan [9, 10]. Shiunko has been proved to improve wound healing by promoting reepithelialization and granulation tissue formation, including angiogenesis [10]; however, there are on reports of the effect of shiunko on lymphangiogenesis. Lymphangiogenesis and angiogenesis are important processes in wound healing [11] and the efficacy of shiunko for the promotion of lymphangiogenesis and also angiogenesis in wound healing may be derived from the effect of components of this herbal medicine.

One of the components of shiunko is Lithospermi Radix (LR, the dried root of *Lithospermum erythrorhizon *Sieb. et Zucc, also called Zicao or Gromwell) that contains several compounds of shikonin and its derivatives, such as deoxyshikonin, acetylshikonin, isobutylshikonin, and others [12]; however, there is no report on shikonin and its derivatives that promote lymphangiogenesis or angiogenesis. In contrast, we found that shikonin and some derivatives have strongly shown to inhibit angiogenesis in *in vitro* and *in vivo *models [9] by suppressing VEGF production, proliferation, and the migration of endothelial cells [13]. These compounds also blocked integrin *α*v*β*3 expression and inhibited B16 melanoma- and tumor necrosis factor-alpha-induced angiogenesis in mice [9]. To find another shikonin derivative that may have an effect on angiogenesis and lymphangiogenesis, deoxyshikonin was examined in this study. Because traditional medicine for lymphangiogenesis treatment has not been explored widely, this study attempted to find a new mechanism of this compound for controlling lymphangiogenesis* in vitro*.

## 2. Materials and Methods

### 2.1. Materials

Deoxyshikonin was purchased from Tokyo Chemical Industry (TCI) (Tokyo, Japan). The compounds were dissolved in dimethylsulfoxide (DMSO) to make a stock solution. Matrigel was purchased from BD Biosciences (San Diego, CA, USA). HIF-1*α* siRNA and the antibodies against HIF-1*α* and PCNA were purchased from Santa Cruz Biotechnology, Inc. (Santa Cruz, CA, USA). Polyclonal rabbit anti-mouse immunoglobulin/HRP, polyclonal goat anti-rabbit immunoglobulin/HRP, and polyclonal swine anti-rabbit immunoglobulins/FITC were purchased from Dako (Glostrup, Denmark). Dynabeads protein G was purchased from Invitrogen (Oslo, Norway). Vectashield mounting medium with DAPI was from Vector Laboratories, Inc. (Burlingame, CA, USA). Rhodamine phalloidin was obtained from Life Technologies (Carlsbad, CA, USA). The culture inserts were from Ibidi (Martinsried, Germany).

### 2.2. Endothelial Cells

Human dermal lymphatic microvascular endothelial cells (HMVEC-dLy) and human dermal microvascular endothelial cells (HMVEC-d) were obtained from Takara Bio Inc. (Shiga, Japan). The cells were primary culture cells. Cells were cultured in Clonetics EGM-2 MV Bullet Kit (Takara Bio) in a humidified atmosphere (5% CO_2_, 95% air). Cells were passaged upon reaching confluence with Trypsin-EDTA solution. To maintain normal growth, the primary cells from 5th to 15th passages were used in the study. 

### 2.3. Proliferation Assay

Cell viability after treatment with various concentrations of deoxyshikonin and shikonin was assessed with a WST-8 cell proliferation assay kit (DOJINDO, Kumamoto, Japan). Cells were cultured in 96-well plates at 37°C. At the time of measurement, 10 *μ*L WST-8 reagent was added to each well and the cells were cultured continuously for 2 h at 37°C in 5% CO_2_. Absorbance was measured at 450 nm to determine cell viability as a percentage. 

### 2.4. Cord Formation on Matrigel

Ninety-six-well plates were coated with 60 *μ*L Matrigel (10 mg/mL) and allowed to polymerize at 37°C. Endothelial cells (8 × 10^3^ cells/well) were seeded on the Matrigel and incubated at 37°C. At each time point, cells were fixed with a 4% paraformaldehyde and stained using Mayer's hematoxylin (Muto Pure Chemical, Tokyo, Japan). The cord network was photographed and cord length was measured using an Angiogenesis Image Analyzer (Kurabo, Osaka, Japan) [14].

### 2.5. Gene Expression Analysis by Real-Time PCR

Briefly, total RNA was extracted from cultured cells on Matrigel using TRIzol reagent (Invitrogen, Carlsbad, CA, USA). For each sample, 0.5 *μ*g of total RNA was reverse transcribed into cDNA using the Prime Script RT reagent kit (Perfect Real Time) (TaKaRa, Dalian, China). Real-time PCR analysis was performed using the LightCycler Nano System (Roche Diagnostics, Mannheim, Germany) using FastStart Essential DNA Green Master (Roche Diagnostics) according to the manufacturer's instructions. GAPDH was used as an internal control. The relative quantification of mRNA expression was calculated as a ratio of the target gene to GAPDH. The primer sequences were as follows: HIF-1*α* sense, 5′-TTTTTCAAGCAGTAGGAATTGGA-3′, and antisense, 5′-GTGATGTAGTAGCTGCATGATCG-3′; VEGF-C sense, 5′-TGCCAGCAACACTACCACAG-3′, and antisense, 5′-GTGATTATTCCACATGTAATTGGTG-3′; VEGF-A sense, 5′-CCTCCGAAACCATGAACTTT-3′, and antisense, 5′-ATGATTCTGCCCTCCTCCTT-3′; VEGF-D sense, 5′-GGAGGAAAATCCACTTGCTG-3′, and antisense, 5′-GCAACGATCTTCGTCAAAC-3′; GAPDH sense, 5′-AGCCACATCGCTCAGACAC-3′, and antisense, 5′-GCCCAATACGACCAAATCC-3′.

### 2.6. Detection of HIF-1*α* and PCNA

To determine protein levels during cord formation on Matrigel, immunoprecipitation and Western blotting were performed as described previously [14]. Cells cultured on Matrigel were washed with PBS and incubated with whole cell lysis buffer (25 mM HEPES pH 7.7, 0.3 M NaCl, 1.5 mM MgCl_2_, 0.2 mM EDTA, 0.1% Triton X-100, 20 mM *β*-glycerophosphate, 1 mM sodium orthovanadate, 1 mM phenylmethylsulfonyl fluoride (PMSF), 1 mM dithiothreitol (DTT), 10 *μ*g/mL aprotinin, and 10 *μ*g/mL leupeptin). The cells including Matrigel were then scrubbed. The collected samples were vigorously vortexed, centrifuged at 14,000 rpm for 10 min, and the supernatant was collected. Immunoprecipitation was carried out by incubating the lysate with HIF-1*α* primary antibody (Santa Cruz Biotechnology) for 16 h at 4°C, followed by 12 h incubation with Dynabeads protein G (Invitrogen). The immunoprecipitates were washed with lysis buffer, resuspended in loading buffer, boiled for 3 min, subjected to SDS-PAGE on 7.5% polyacrylamide gels, and transferred to a PVDF membrane. The primary antibody used was for HIF-1*α* and the secondary was polyclonal goat anti-rabbit immunoglobulin/HRP. The bands were detected using an immunochemiluminescence method. PCNA was used as the loading control.

### 2.7. Immunofluorescence Microscopy

Cells were seeded onto a cover slip coated with Matrigel (10 mg/mL). Cells were incubated with or without deoxyshikonin-containing medium. At the time of the experiment, the attached cells were washed with PBS, fixed with 4% paraformaldehyde (10 min), and washed and permeabilized (5 min) with 0.1% Triton X-100 in PBS. Samples were blocked with 1% BSA in PBS followed by incubation for 30 min with HIF-1*α* rabbit polyclonal primary antibody (Santa Cruz Biotechnology). After incubation with primary antibody, cells were washed in 0.2% Triton X-100 in PBS and then incubated with polyclonal swine anti-rabbit immunoglobulins/FITC (Dako) as a secondary antibody and Rhodamine phalloidin (Life Technologies) for 20 min. After washing, Vectashield mounting medium for DAPI staining (Vector, Burlingame, CA, USA) was added to the cells. Florescence images were captured using a Leica TCS SP5 microscope. 

### 2.8. siRNA Transfection

Proliferating HMVEC-dLy was transfected with control siRNA or siRNA against HIF-1*α* (Santa Cruz Biotechnology) at a final concentration of 6 nM using Lipofectamine RNAiMAX reagents (Invitrogen). After transfection, the cells were grown for 18 h at 37°C in 5% CO_2_ and trypsinized. The transfected cells combined with or without deoxyshikonin were seeded on Matrigel-coated dishes. At each time point, cells were employed for real-time PCR and cord formation assays.

### 2.9. Wound-Healing Assay

To investigate the potential wound-healing ability with deoxyshikonin treatment, a modified scratch assay was performed, creating gaps of precisely defined width. Culture inserts from Ibidi (Martinsried, Germany) were used in this study. This insert creates a cell-free gap (approximately 500–600 *μ*m) [15]. Seventy microliters of cell suspension (1.8 × 10^5^ cells/mL) were added to each well of the Ibidi culture insert. Cells were incubated at 37°C for 48 h until the cells were confluent and then the culture inserts were removed to create the gap and to allow cell migration to fill it over time. Cell migration into the gap was monitored by inverted microscopy and photographed at each time point. The distance between one side of the gap and the other can be measured by comparing the image from time 0 h to the last time point at 24 h. The distance between each gap closer was measured using Leica LAS EZ software and then calculated as the migration distance (mm). 

### 2.10. Statistical Analysis

Statistical analysis was performed using Dunnett's method. *P* < 0.05 was considered to be significant.

## 3. Results

### 3.1. Deoxyshikonin Enhanced Cord Formation of HMVEC-dLy and HMVEC-d on Matrigel

Shikonin and some shikonin derivatives have been reported to inhibit angiogenesis [13, 16]. To find new effect of compounds that affects lymphangiogenesis and angiogenesis, deoxyshikonin was selected for use in the present study. The nontoxic dose of 0.8 *μ*M deoxyshikonin was used to see the effect on cord formation ability of human lymphatic endothelial cells (HMVEC-dLy) and human dermal microvascular endothelial cells (HMVEC-d) (Figure 2). The cells underwent the cord formation assay and were photographed (Figures 2(a) and 2(c)) at 2 to 6 h after seeding on Matrigel. Cord length was measured by an Angiogenesis Image Analyzer and plotted as a percentage (Figures 2(b) and 2(d)). Deoxyshikonin significantly promoted cord formation ability by 64% and 28% from the control in HMVEC-dLy and HMVEC-d at 6 h of incubation, respectively (Figures 2(b) and 2(c)). This is a newly discovered effect of deoxyshikonin, which promoted to lymphangiogenesis and angiogenesis in an *in vitro* model and showed the opposite effect to shikonin (data not shown).

Because knowledge about the mechanism of lymphangiogenesis and treatment with natural compounds has not been explored sufficiently, we decided to further confirm the possible mechanism of this natural compound, deoxyshikonin, on lymphangiogenesis.

The time that showed a significant change in cord formation networks, 6 h of deoxyshikonin treatment in HMVEC-dLy, was chosen for use in further experiments. 

### 3.2. Deoxyshikonin Dominantly Increased VEGF-C mRNA Level in HMVEC-dLy While Forming Cords on Matrigel

Endothelial cells are the target of VEGF-C, -A, and -D in lymphangiogenesis induction [4]. In addition, endothelial cells themselves can express VEGF mRNA and protein levels after stimulation [17]. The cord formation ability of HMVEC-dLy, which was enhanced by deoxyshikonin (Figures 2(a) and 2(b)), may occur as a result of the increase of VEGFs.

We further examined the effect of deoxyshikonin on the expression of VEGF-C, VEGF-A, and VEGF-D mRNA during cord formation of HMVEC-dLy. Real-time PCR was used to determine transcription levels of these genes. Endothelial cells were seeded on Matrigel and incubated with 0.8 *μ*M deoxyshikonin for 6 h. The mRNA was collected and subjected to real-time PCR (Figure 3(b)). The cord formation assay was also performed for comparison at the same time of incubation (Figure 3(a)). The results showed that deoxyshikonin significantly increased mRNA expression levels of VEGF-C and VEGF-A in HMVEC-dLy by 0.42-fold and 0.32-fold when compared with their control, respectively (Figure 3(b)). These increases also correlated with the increase of cord formation of endothelial cells at the same time of treatment (Figure 3(a)); however, VEGF-D mRNA levels were expressed at very low levels (Figure 3(b)). This indicated that deoxyshikonin-induced cord formation networks in HMVEC-dLy were involved in the induction of VEGF-C and VEGF-A mRNA levels, which had a greater potential effect on the increase of the VEGF-C mRNA level than the VEGF-A mRNA level (Figure 3(b)). 

VEGF-C is the main vascular endothelial growth factor important for lymphangiogenesis [18, 19]. As the next step, we studied the possible mechanisms of deoxyshikonin induction in lymphangiogenesis* in vitro* by using HMVEC-dLy. 

### 3.3. Deoxyshikonin Regulates HIF-1*α* at Transcriptional, Posttranscriptional, and Functional Levels in HMVEC-dLy during Cord Formation on Matrigel

HIF-1*α* plays a certain role in lymphangiogenesis by closely correlating with lymphatic expression of VEGF-C in cancers, wound healing, and inflammation [7, 20, 21], and we found that deoxyshikonin upregulated the VEGF-C mRNA level during lymphangiogenesis *in vitro* (Figure 3(b)). In addition, the function of HIF-1 can be regulated by several stimuli under normoxic conditions [4, 5]. Deoxyshikonin, which promoted cord formation of HMVEC-dLy in this study, may correlate with VEGF-C mRNA expression and HIF-1*α* regulation.

To determine the effect of deoxyshikonin on HIF-1*α* regulation, real-time PCR, immunoprecipitation/Western blotting, and immunofluorescence microscopy were performed to see the expression of HIF-1*α* mRNA, HIF-1*α* protein, and also the activation of HIF-1*α*, respectively. HMVEC-dLy were seeded on Matrigel, cultured with or without 0.8 *μ*M deoxyshikonin-containing medium, and left to form cord networks for 6 h. Then, mRNA and protein were collected to measure mRNA and protein expression levels.  Figure 3(c) shows that the HIF-1*α* mRNA level was increased 0.58-fold that of the control (Figure 3(c)) and the HIF-1*α* protein level also significantly increased after deoxyshikonin treatment (Figure 3(d)). The nuclear translocation of HIF-1*α* was also determined (Figure 3(e)). Cells were seeded on Matrigel-coated slides and then treated with or without 0.8 *μ*M deoxyshikonin for 6 h. Immunofluorescence microscopy was performed and photographed (Figure 3(e)). HIF-1*α* is a cytoplasmic protein. During activation, HIF-1*α* will dimerize with HIF-1*β* and then translocate to the nucleus to give the active transcription factor of HIF-1. In our results, HMVEC-dLy treated with deoxyshikonin showed the accumulation of HIF-1*α* inside the nucleus, which indicated the activation of HIF-1*α* transcription factor. These results conclude that deoxyshikonin induced cord formation of HMVEC-dLy and was involved in the regulation of HIF-1*α* at the transcriptional, posttranscriptional, and functional levels. 

### 3.4. Deoxyshikonin-Induced VEGF-C mRNA Expression and Cord Formation of HMVEC-dLy via HIF-1*α* Regulation

To see whether HIF-1*α* controls VEGF-C mRNA expression during cord formation of deoxyshikonin-treated cells, siHIF-1*α* transfection was used in this study. siCont- and siHIF-1*α*-transfected cells were seeded on Matrigel and incubated with or without 0.8 *μ*M deoxyshikonin for 2–6 h. At each time point, cells underwent a cord formation assay (Figure 4(c)) and mRNA was collected to perform real-time PCR (Figures 4(a) and 4(b)). The results showed that HMVEC-dLy, which was transfected with siHIF-1*α*, successfully suppressed HIF-1*α* mRNA expression throughout the experiment (Figure 4(a)). During incubation, in the absence of deoxyshikonin, VEGF-C mRNA expression was significantly decreased in HIF-1*α* knockdown cells, by 0.25-fold, 0.5-fold, and 0.2-fold at 2, 4, and 6 h, respectively (Figure 4(b)) and these decreases also correlated with the significant decrease in the length of cord formation by the HIF-1*α* knockdown cells, by 38%, 56%, and 46% at 2, 4, and 6 h, respectively, (Figure 4(c)). These results indicated that HIF-1*α* controlled VEGF-C mRNA expression and cord formation ability in HMVED-dLy. In addition, deoxyshikonin treatment in siCont-transfected cells significantly increased HIF-1*α* and VEGF-C mRNA expression at 6 h (Figures 4(a) and 4(b)) when compared with their control groups. These increases also correlated with the significantly increased cord formation at the same incubation time (6 h) (Figure 4(c)). Interestingly, deoxyshikonin treatment of HIF-1*α* knockdown cells did not restore VEGF-C mRNA expression (Figure 4(b)) and cord formation (Figure 4(c)) to the control levels but only slightly increased their levels when compared to untreated HIF-1*α* knockdown cells.

These results indicated that deoxyshikonin promoted VEGF-C mRNA expression and the cord formation ability of HMVEC-dLy, mainly via HIF-1*α*-dependent regulation.

### 3.5. Deoxyshikonin Promoted Wound Healing *In Vitro*

We therefore succeeded in proving the mechanism of deoxyshikonin on the cord formation of HMVEC-dLy, which was involved in the regulation of HIF-1*α* and VEGF-C mRNA expression. As the next step, we assessed the potential of deoxyshikonin-induced lymphangiogenesis for clinical applications such as wound-healing treatment, because the promotion of lymphangiogenesis can improve wound-healing [22]. We performed a wound healing assay using culture inserts from Ibidi to create a cell-free gap and measured the gap distance (migration distance) using Leica LAS EZ software. The result showed that incubating the cells with 0.8 *μ*M deoxyshikonin promoted the migration of HMVEC-dLy by significantly inducing cell filling of the gap when compared to the control at 24 h of incubation (Figures 5(a) and 5(b)). Some of the effect of deoxyshikonin on apparent cell migration might be a contribution from cell proliferation. The proliferation assay (Figure 5(c)) confirmed that at a deoxyshikonin concentration of 0.8 *μ*M, the cells filling the gap of the wound did not arise from cell proliferation. However, at higher concentrations (1.6 and 3 *μ*M), deoxyshikonin significantly induced proliferation of HMVEC-dLy. This result indicated that deoxyshikonin could be used to improve wound healing by inducting lymphatic endothelial cell migration and lymphangiogenesis. 

## 4. Discussion

**Figure 1:**
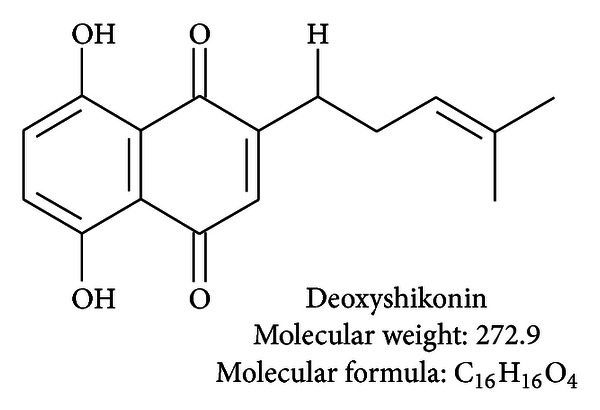
Chemical structure of deoxyshikonin.

In this study, we used primary endothelial cells, human dermal lymphatic microvascular endothelial cells (HMVEC-dLy), to investigate a new effect of deoxyshikonin (Figure 1), on lymphangiogenesis *in vitro* by comparing them with human dermal microvascular endothelial cells (HMVEC-d). We continued to explore the possible mechanism by investigating the expression of important genes involved in cord formation networks of lymphatic endothelial cells after treatment with deoxyshikonin. 

Previous reports found that deoxyshikonin has antifungal [23] and antitumor activities [24, 25], but there was no evidence of a lymphangiogenesis or angiogenesis effect. Shikonin and some derivative forms such as acetylshikonin, isobutyroylshikonin, and *β*-hydroxyisovalerylshikonin are already known to have an antiangiogenesis effect on an *in vivo* and *in vitro *model and the controlling molecules are known [9, 13, 16]; however, the effect of shikonin and its derivatives, including deoxyshikonin, on lymphangiogenesis has not been discovered.

**Figure 2:**
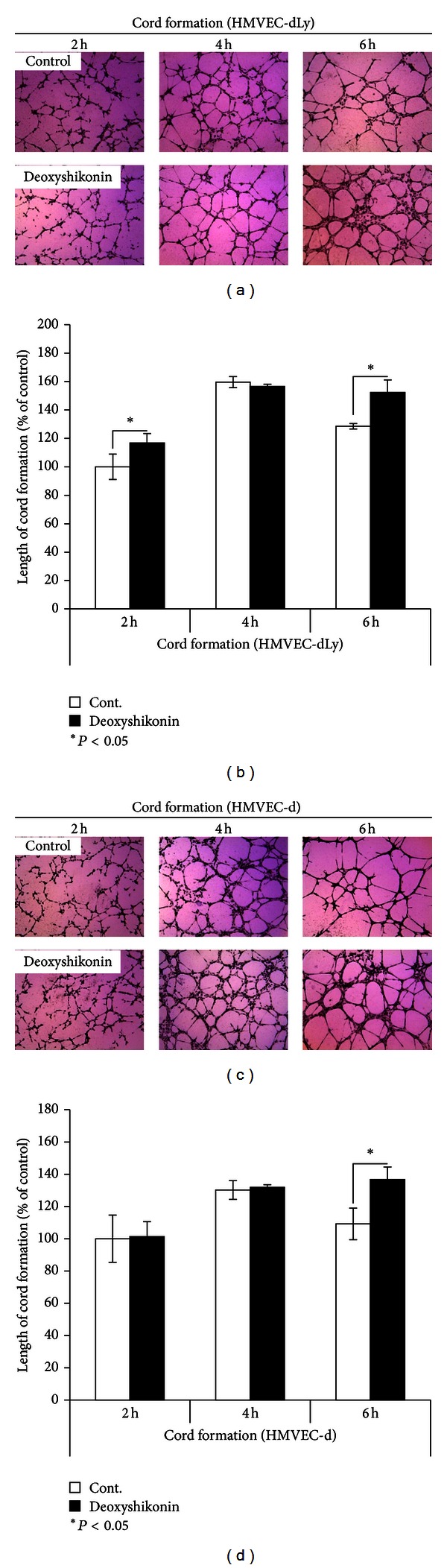
Effects of deoxyshikonin on cord formation of HMVEC-dLy and HMVEC-d on Matrigel. (a), (c) Photographs of cord formation of HMVEC-dLy and HMVEC-d on Matrigel after incubation with or without 0.8 *μ*M deoxyshikonin at 2 to 6 h (at ×400 magnification). (b), (d) The relative length of cords was measured using an Angiogenesis Image Analyzer. Data are the mean ± SD (*n* = 3); *P < 0.05, **P < 0.01 compared with the control.

The nontoxic dose of deoxyshikonin was confirmed (Figure 5(c)) and selected in a proliferation assay before performing the experiments. Our results show for the first time that deoxyshikonin has a potential to promote lymphangiogenesis and angiogenesis in an* in vitro *model, which interestingly showed opposite effects with shikonin (See Supplementary Figures 1(b) and 1(c) available online at http://dx.doi.org/10.1155/2013/148297). We showed for the first time that deoxyshikonin has a prolymphangiogenesis (Figures 2(a) and 2(b)) as well as a proangiogenesis effect *in vitro* (Figures 2(c) and 2(d)).

**Figure 3:**
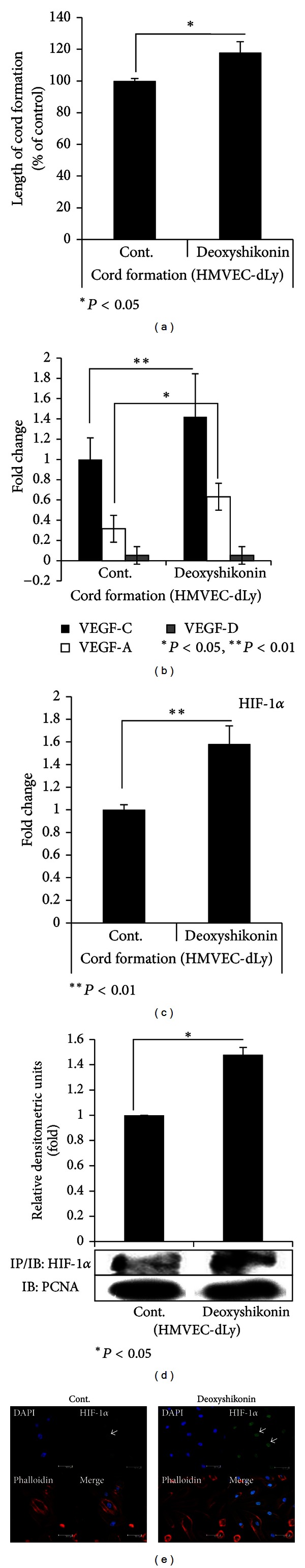
Effect of deoxyshikonin on VEGF-C, -A, and -D mRNA levels and regulation of HIF-1*α* during cord formation of HMVEC-dLy on Matrigel. Cells were exposed with or without 0.8 *μ*M deoxyshikonin for 6 h and then underwent experiments. (a) The relative length of cords was measured using an Angiogenesis Image Analyzer. (b), (c) VEGF-C, -A, and -D and also HIF-1*α* mRNA levels were detected by real-time PCR. (d) The HIF-1*α* protein level was determined by immunoprecipitation and Western blotting. The results were analyzed by scanning and Scion Image software. (e) HIF-1*α* nuclear translocation as determined by immunofluorescence microscopy. Similar results were obtained in three independent experiments; *P < 0.05, **P < 0.01 compared with their control.

The processes of the cord formation of endothelial cells after seeding on Matrigel, which mimics the extracellular matrix [26], include the promotion of cell adhesion, survival, and migration, including cell proliferation for sprouting and finding each other and maintaining the formation cord networks [27], and VEGFs such as VEGF-C, VEGF-A, and VEGF-D are widely known to induce lymphangiogenesis and angiogenesis *in vitro *and* in vivo* by enhancing these processes [28]. Generally, endothelial cells are the target, not the main source of VEGFs; however, it has been also demonstrated that human dermal microvascular endothelial cells themselves can express mRNA and release an amount of these growth factors [17, 28]. We found that deoxyshikonin significantly increased the expression of VEGF-C mRNA and VEGF-A mRNA in HMVEC-dLy but had no effect on VEGF-D (Figure 3(b)). The increase of VEGF-C and VEGF-A correlated with the significant induction of the cord formation of HMVEC-dLy at the time of deoxyshikonin treatment (Figure 3(a)). Endothelium-derived VEGF can induce neovascularization through proliferation, and increase the migration of dermal microvascular cells [29]. The increase of VEGF-C and VEGF-A mRNA expression after deoxyshikonin treatment could proceed to protein products, be secreted, and then interact with specific membrane receptors of endothelial cells displaying tyrosine kinase activity [17]. Binding of VEGF-A with VEGFR-2 and binding of VEGF-C with VEGFR-2 and VEGFR-3 promote the cord formation of HMVEC-dLy on Matrigel (Figure 3(a)). Interestingly, the results (Figure 3(b)) show that the mRNA levels of VEGF-C after deoxyshikonin treatment were high when compared with VEGF-A. As VEGF-C acts as a key growth factor in physiological lymphangiogenesis and was found to promote the activation of VEGF-3, a specific receptor expressed in lymphatic endothelium [18, 19], we focused our study on the mechanism of deoxyshikonin on lymphangiogenesis *in vitro*. 

HIF-1 is an oxygen-regulated transcriptional factor that plays a role in tumor lymphangiogenesis, wound healing and inflammation by regulating the lymphatic expression of VEGF-C [7, 20, 21]. In addition, HIF-1-mediated pathways also promote or repress the transcription of a broad range of genes that are involved in maintaining biological homeostasis, such as influencing metabolic adaptation, the innate immune response, cell survival, and apoptosis [4, 5]. In hypoxia, HIF-1*α* protein persists and the HIF-1*α*/*β* complex stimulates VEGF release in almost all cell types. Under normoxia, HIF-1*α* protein is subjected to ubiquitin-dependent degradation [4]; however, HIF-1*α* is also expressed and functions in response to stimulation by several growth factors by the mechanism different from the hypoxic condition [5]. 

In this study, we performed experiments under normoxic conditions. For the first time we found that deoxyshikonin regulated HIF-1*α* at transcriptional, posttranscriptional, and functional levels (Figures 3(c), 3(d), and 3(e)) during cord formation of HMVEC-dLy (Figure 3(a)). Similar recent reports mentioned that several nonhypoxic effectors and signaling pathways have been proven to enhance HIF-1*α* levels through the activation of regulative mechanisms distinct from protein stabilization. Some of these stimuli also regulate HIF-1*α* at the transcriptional, posttranscriptional, or translational level or additionally influence posttranslational modifications, including the functions of HIF-1*α* protein [4, 5]. For example, lipopolysaccharides (LPS) and cytokines activate the nuclear factor-*κ*B (NF-*κ*B) signaling pathway promoting HIF-1*α* transcription [30], whereas some growth factors such as epithelial growth factors (EGF), fibroblast growth factor 2 (FGF2), and insulin-like growth factor (IGF) enhance the translation of HIF-1*α* protein [31]. In addition, loss of function of tumor suppressors (such as p53, PTEN, and VHL) and gain of function of oncogenes (such as AKT, MYC, mTOR, PI3 K, RAF, and RAS) also regulate different steps that lead to the activation of HIF function [31, 32]. 

**Figure 4:**
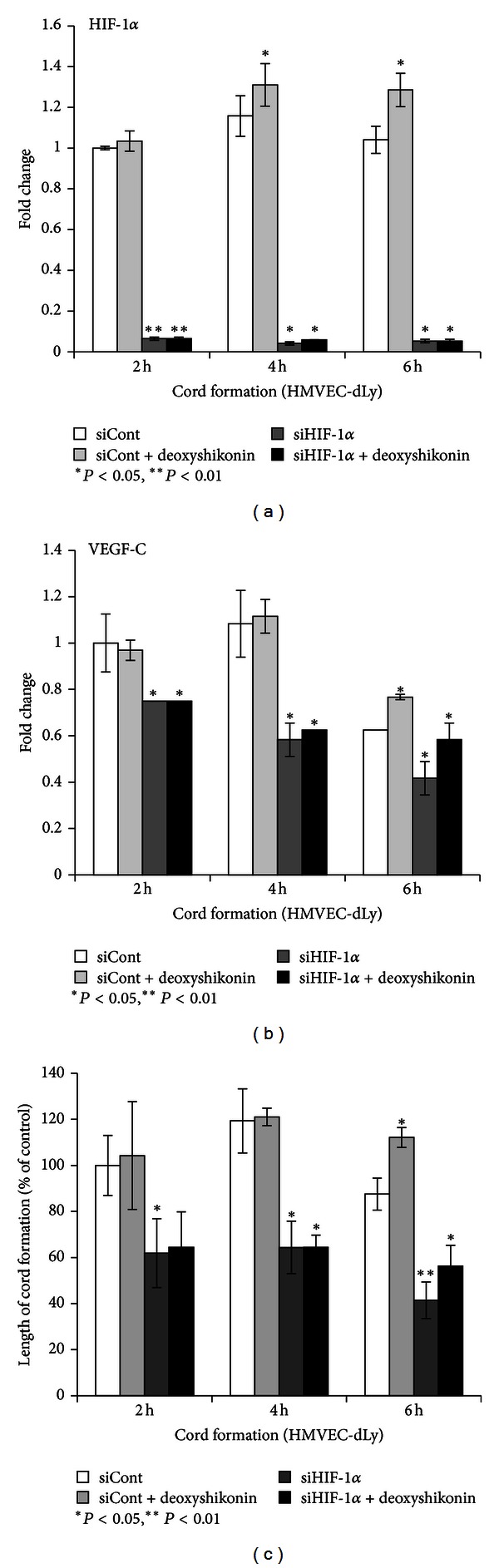
Deoxyshikonin regulates VEGF-C mRNA levels and cord formation of HMVEC-dLy via HIF-1*α*. After transfection with control siRNA or siRNA for HIF-1*α*, HMVEC-dLy cells were seeded on Matrigel and incubated with or without 0.8 *μ*M deoxyshikonin for 2–6 h, and then cells were subjected to real-time PCR and cord formation assay. (a) HIF-1*α* mRNA levels. (b) VEGF-C mRNA levels. (c) Relative length of cord formations. Similar results were obtained in three independent experiments; *P < 0.05, **P < 0.01 compared with the control.

Deoxyshikonin might contribute to the signaling pathways mentioned above, enhance HIF-1*α* mRNA/protein expression and activate nuclear translocation (Figures 3(c), 3(d), and 3(e)); however, we did not prove the effect of deoxyshikonin on the signal transduction pathway in this study. Once in the nucleus, deoxyshikonin could promote HIF-1*α* and HIF-1*β* subunit interaction and bind to specific DNA sequences targeted by HIF, known as hypoxia response elements (HREs), which are composed of 5′-RCGTG-3′, leading to the stimulation of VEGF release, especially VEGF-C, which induced cord formation of HMVEC-dLy (Figures 3(a) and 3(b)). Successful suppression of the HIF-1*α* gene using siRNA transfection confirmed that HIF-1*α* regulated VEGF-C mRNA expression and the cord formation ability of HMVEC-dLy on Matrigel (Figure 4).

Although VEGF mRNA, including VEGF-C mRNA expression, can be upregulated by HIF-1, several transcription factors such as AP-1, Sp-1, and NF-*κ*B also induce VEGF expression by binding to the promoter to initiate and activate the transcription of the VEGF gene directly [6]. We also proved this by using deoxyshikonin treatment in HIF-1*α* knockdown cells and found that deoxyshikonin could not recover VEGF-C mRNA expression and the cord formation of HMVEC-dLy back to the control level but only slightly increased when compared with HIF-1*α* knockdown cells alone (Figure 4). This result indicated that deoxyshikonin induced VEGF-C mRNA expression and the cord formation of HMVEC-dLy, mainly via HIF-1*α*-dependent regulation, and may also contribute to HIF-1*α*-independent regulation; however, the details of these mechanisms still need to be further investigated.

**Figure 5:**
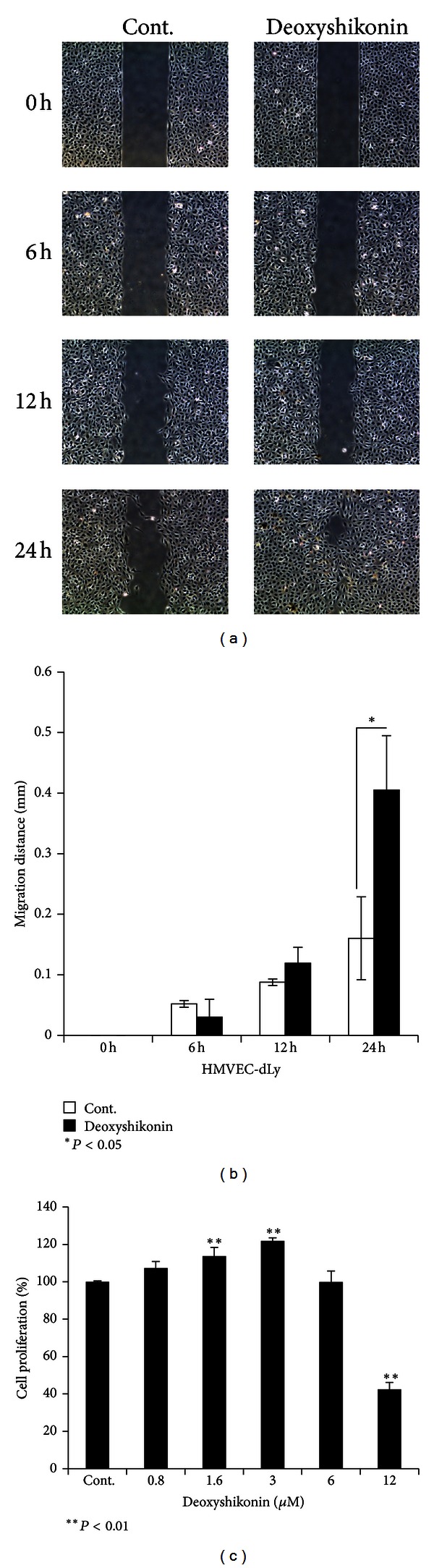
Deoxyshikonin promoted wound healing *in vitro* by inducing the HMVEC-dLy migration ability. After growing the HMVEC-dLy in culture inserts that create a cell-free gap, migration of cells to fill the gap was monitored at regular intervals. (a) Photograph of cell migration into the gap from 0 to 24 h in deoxyshikonin treatment and control group. (b) The migration ability of the cells was measured using Leica LAS EZ software and the migration distances calculated (mm). (c) The effects of deoxyshikonin on cell proliferation were determined by a proliferation assay and the data are plotted as percentages of control cell proliferation. Data are the mean ± SD (*n* = 3); *P < 0.05, **P < 0.01 compared with the control.

The promotion of lymphatic vessel generation improved wound function to maintain normal tissue pressure by draining protein-rich lymph from the interstitial space and facilitate the delivery of cells that mediate the immune response [22]. In this study we proved that deoxyshikonin promoted lymphangiogenesis (Figure 2) and also wound healing *in vitro* by facilitating the migration of HMVEC-dLy into the wound gap (Figure 5), which indicated that deoxyshikonin could be developed for use in wound-healing treatment. However, wound healing is a complicated biological process as it involves the interactions of multiple cell types, various cytokines, growth factors, their mediators, and extracellular matrix proteins [11], and the details of deoxyshikonin in wound healing require further proof *in vitro* and also *in vivo.*

In conclusion, we discovered a new effect of deoxyshikonin, which is included in shiunko as a typical Kampo drug ointment used for the treatment of wound healing in Japan, that enhanced cord formation of HMVEC-dLy via HIF-1*α*-controlled VEGF-C mRNA regulation and also promoted wound healing in an *in vitro* model. This finding may offer new therapeutic options for using deoxyshikonin compounds that modulate HIF-1*α* and VEGF-C under nonhypoxic conditions in wound healing and other lymphatic diseases.

## Supplementary Material

Supplementary Figure: Effect of shikonin on cord formation in HMVEC-dLy compared with deoxyshikonin.
