# Supplementary material for: Enhancement of Lymphangiogenesis In Vitro via the Regulations of HIF-1α Expression and Nuclear Translocation by Deoxyshikonin
Source: Evid Based Complement Alternat Med. 2013 Apr 22;2013:148297. doi: 10.1155/2013/148297 (PMC3664343; doi:10.1155/2013/148297)

**Supplementary Fig. 1.**

**Material and methods (Supplementary Fig. 1.)**

Shikonin was obtained from Sigma Chemicals (St. Louis, MO). The compounds were dissolved in dimethylsulfoxide (DMSO) to make stock solutions. Shikonin at a non-toxic dose of 0.8 μM was selected for comparison to deoxyshikonin in cord formation assays by HMVEC-dLy after 6 h incubation on Matrigel.

**Results (Supplementary Fig. 1.)**

A non-toxic dose (0.8 μM) of shikonin significantly suppressed cord formation of HMVEC-dLy by 19% 6 h after seeding on Matrigel. The opposite effect was found for deoxyshikonin, which significantly promoted cord formation by 18% over control HMVEC-dLy at the same treatment time and dose as shikonin. This result confirmed that deoxyshikonin promoted lymphangiogenesis in the *in vitro* model and showed the opposite effect to shikonin.

**Figure legend (Supplementary Fig. 1.)**

Effect of shikonin on cord formation in HMVEC-dLy compared with deoxyshikonin. **A.** Chemical structures of shikonin and deoxyshikonin. **B.** Images of cord formation by HMVEC-dLy on Matrigel after 6h incubation with or without 0.8 μM shikonin and deoxyshikonin (at x400 magnification). **C.** The relative lengths of cord formation were measured using an Angiogenesis Image Analyzer. Data are the mean ± SD (n = 3); **P*<0.05, ***P*<0.01 compared with the control.


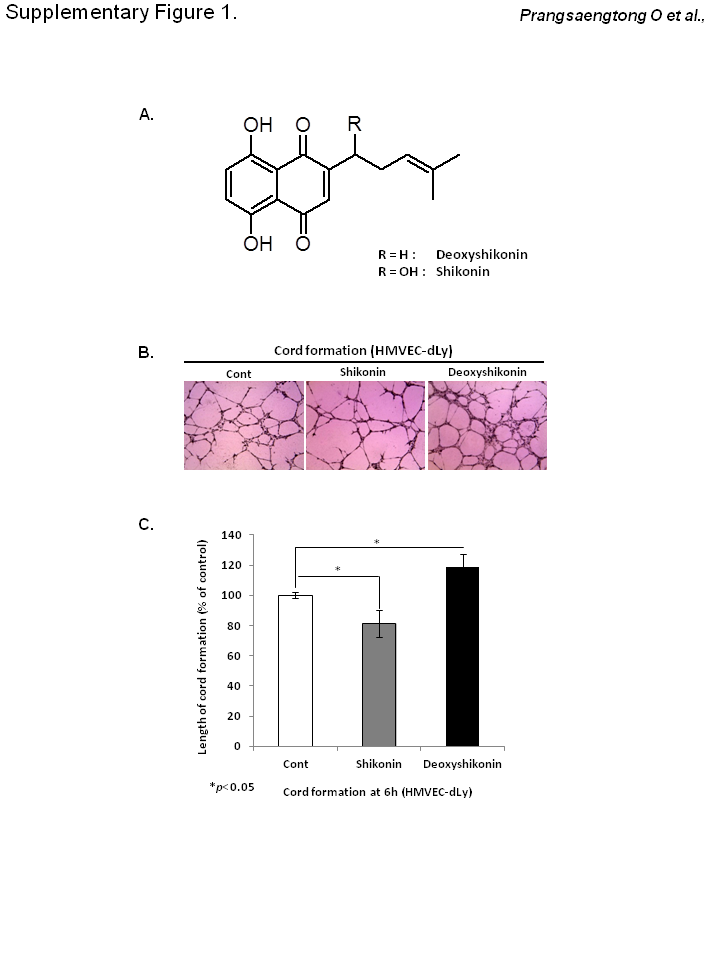

Supplement: Supplementary file 1 — Supplementary Figure: Effect of shikonin on cord formation in HMVEC-dLy compared with deoxyshikonin. [file 148297.f1.doc]
